# Metabolic Engineering of *Escherichia coli* for *para*-Amino-Phenylethanol and *para*-Amino-Phenylacetic Acid Biosynthesis

**DOI:** 10.3389/fbioe.2018.00201

**Published:** 2019-01-04

**Authors:** Behrouz Mohammadi Nargesi, Georg A. Sprenger, Jung-Won Youn

**Affiliations:** Institute of Microbiology, University of Stuttgart, Stuttgart, Germany

**Keywords:** *Escherichia coli*, aromatic amines, *para-*amino-phenylethanol, *para*-amino-phenylacetic acid, *para*-amino-phenylacetaldehyde, phenylpyruvate decarboxylase

## Abstract

Aromatic amines are an important class of chemicals which are used as building blocks for the synthesis of polymers and pharmaceuticals. In this study we establish a *de novo* pathway for the biosynthesis of the aromatic amines *para-*amino-phenylethanol (PAPE) and *para-*amino-phenylacetic acid (4-APA) in *Escherichia coli*. We combined a synthetic *para*-amino-l-phenylalanine pathway with the fungal Ehrlich pathway. Therefore, we overexpressed the heterologous genes encoding 4-amino-4-deoxychorismate synthase (*pabAB* from *Corynebacterium glutamicum*), 4-amino-4-deoxychorismate mutase and 4-amino-4-deoxyprephenate dehydrogenase (*papB* and *papC* from *Streptomyces venezuelae*) and ThDP-dependent keto-acid decarboxylase (*aro*10 from *Saccharomyces cerevisiae*) in *E. coli*. The resulting *para*-amino-phenylacetaldehyde either was reduced to PAPE or oxidized to 4-APA. The wild type strain *E. coli* LJ110 with a plasmid carrying these four genes produced (in shake flask cultures) 11 ± 1.5 mg l^−1^ of PAPE from glucose (4.5 g l^−1^). By the additional cloning and expression of *feaB* (phenylacetaldehyde dehydrogenase from *E. coli*) 36 ± 5 mg l^−1^ of 4-APA were obtained from 4.5 g l^−1^ glucose. Competing reactions, such as the genes for aminotransferases (*aspC* and *tyrB*) or for biosynthesis of L-phenylalanine and L-tyrosine (*pheA, tyrA*) and for the regulator TyrR were removed. Additionally, the *E. coli* genes *aroFBL* were cloned and expressed from a second plasmid. The best producer strains of *E. coli* showed improved formation of PAPE and 4-APA, respectively. Plasmid-borne expression of an aldehyde reductase (*yahK* from *E. coli*) gave best values for PAPE production, whereas *feaB*-overexpression led to best values for 4-APA. In fed-batch cultivation, the best producer strains achieved 2.5 ± 0.15 g l^−1^ of PAPE from glucose (11% C mol mol-1 glucose) and 3.4 ± 0.3 g l^−1^ of 4-APA (17% C mol mol^−1^ glucose), respectively which are the highest values for recombinant strains reported so far.

## Introduction

**Figure 1:**
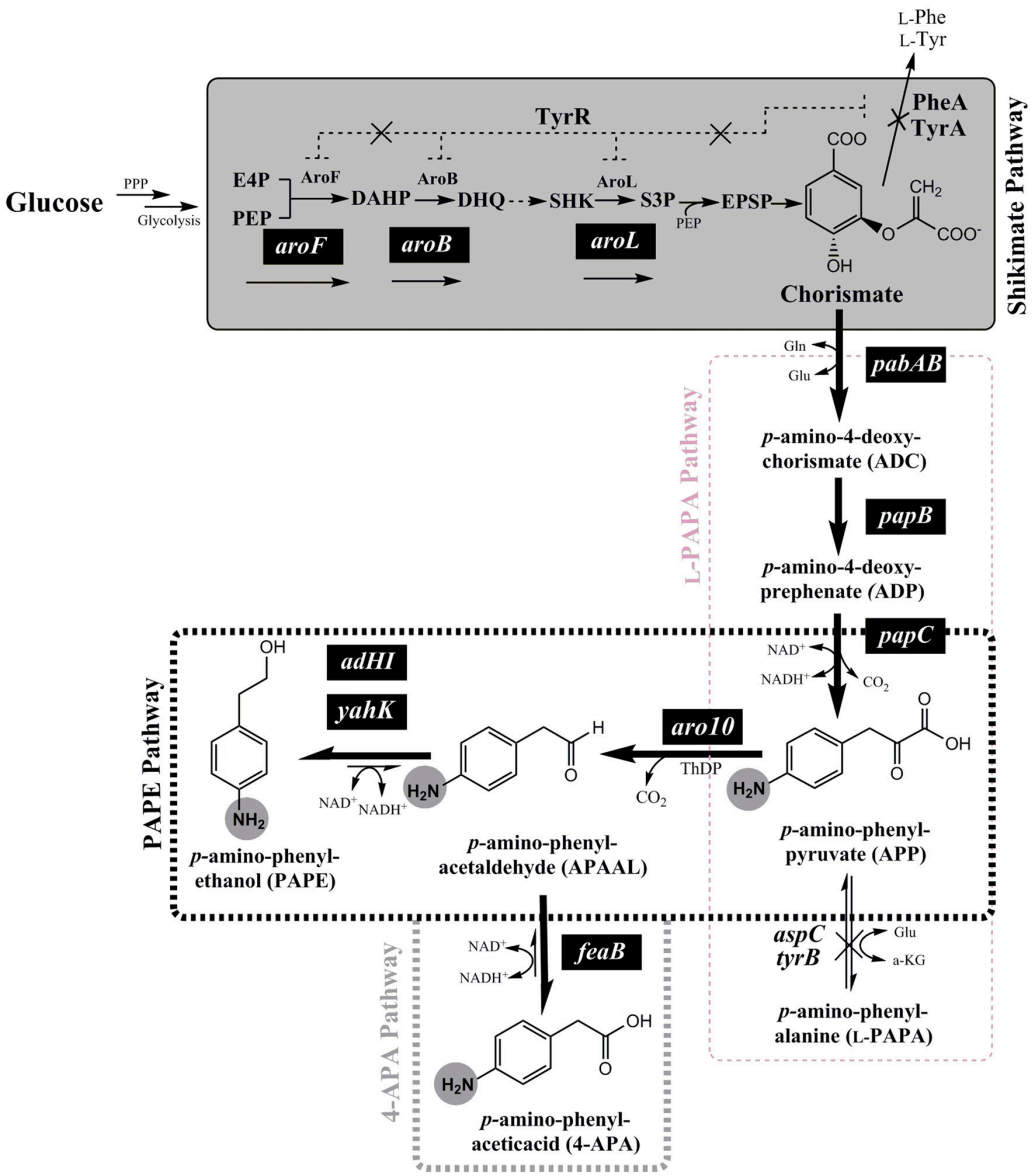
Schematic overview of the *de-novo* biosynthesis pathway of *para-*amino phenylethanol (PAPE) or *para-*amino phenylacetic acid (4-APA) via expanded shikimate pathway in *E. coli* strains from glucose. The genes expressed from plasmids are indicated by white letters on black backgrounds (*pabAB, papB, papC, aro10, aroF, aroB, aroL, adHI, adHII, yahK*, or *feaB*). Enzymes and molecules are abbreviated as follows: G6P, glucose 6-phosphate; F6P, fructose 6-phosphate; F1.6-BP, fructose 1,6-bisphosphate; G3P, glyceraldehyde 3-phosphate; DHAP, dihydroxyacetone phosphate; PPP, Pentose phosphate pathway; E4P, erythrose 4-phosphate; PEP, phosphoenolpyruvic acid; DAHP, 3-deoxy-D-arabinoheptulosonate 7-phosphate; DHQ, dehydroquinate; SHK, shikimic acid; S3P, shikimate 3-phosphate; *aroF*, DAHP synthase; *aroB*, dehydroquinate synthase; *aroL*, shikimate kinase I; *pabAB, p*-amino-4-deoxychorismate (ADC) synthase; *papB*, ADC mutase; *papC, p*-amino-4-deoxyprephenate dehydrogenase; *tyrB*, aromatic aminotransferase; *aspC*, aspartate aminotransferase; *aro10*, ThDP-ketoacid decarboxylase; *ADHI* and *ADHII*, alcohol dehydrogenase I and II; *yahK*, aldehyde reductase; *feaB*, phenylacetaldehyde dehydrogenase.

Aromatic amines (AA) are an important class of chemicals of which several are already used and characterized for different industrial applications. These chemicals can be used for the manufacturing of dyes, pesticides, drugs, plastics, semi-conductive or conductive polymers, and other industrial products (Lawrence, 2004; Vogt and Gerulis, 2005; Sousa et al., 2013; Arora, 2015; Masuo et al., 2016; Tateyama et al., 2016; Tsuge et al., 2016; Kawasaki et al., 2018). AA contain at least one benzene ring and one amino group attached directly to a benzene ring and are usually derived from petroleum-based substrates (Vogt and Gerulis, 2005); they can also be found in Nature. The AA *para*-aminobenzoic acid (PABA) is an intermediate of the folate biosynthesis pathway (Koma et al., 2014; Kubota et al., 2016) while *ortho*-aminobenzoic acid (anthranilic acid) is the first intermediate of the l-tryptophan biosynthesis pathway. The non-proteinogenic aromatic amino acid *para*-amino-l-phenylalanine (l-PAPA) is an important building block for biosynthesis of antibiotics like chloramphenicol (Brown et al., 1996; Chang et al., 2001; He et al., 2001) or of pristinamycin (Blanc et al., 1997). The AA *para*-amino-phenylethanol (PAPE) (see Figure 1), an analog of 2-phenylethanol which has a rose-like odor (Etschmann et al., 2002), is an important building block for chemical syntheses. PAPE can be used for the synthesis of aromatic polyamides (Masuo et al., 2016), polymers or copolymers (Li et al., 1999; Xu et al., 2008), and for the surface functionalization of graphene (Yadav and Cho, 2013). PAPE can also be used for the synthesis of the overactive bladder treatment drug Myrbetriq® (Sacco and Bientinesi, 2012). A classical synthesis of aromatic amines is performed by nitroreduction of aromatic compounds in the presence of acidic compounds and a reducing agent, such as solid metals (Vogt and Gerulis, 2005; Schul'tsev, 2011; Froidevaux et al., 2016).

Due to the fast growing demand for polymers and “super-engineered” plastics (Masuo et al., 2016; Tateyama et al., 2016; Tsuge et al., 2016; Kawaguchi et al., 2017; Kawasaki et al., 2018), PAPE, *p*-amino-phenylacetate (4-APA) and other AAs are considered as important substrates for the synthesis of materials for novel technological applications. So they have become promising candidates for the replacement of metal or ceramic materials (Kawasaki et al., 2018). Hence, an annual global production of about 100,000 tons of AA with an expected market size of several hundred millions US dollars have been estimated (Masuo et al., 2016). Due to the high-value and industrial importance, various approaches to synthesize aromatic amines by biological routes have been started. Thus, PABA (Koma et al., 2014; Kubota et al., 2016), l-PAPA (Mehl et al., 2003; Masuo et al., 2016; Tateyama et al., 2016; Konishi et al., 2017; Mohammadi Nargesi et al., 2018), 4-aminocinnamic acid (Suvannasara et al., 2014; Konishi et al., 2016; Kawasaki et al., 2018), 4-aminohydrocinnamic acid (Tateyama et al., 2016; Kawasaki et al., 2018), have already been produced with genetically engineered *Corynebacterium glutamicum* or *E. coli* strains. More specifically for the present study, Takaya and coworkers recently described an approach to produce PAPE or 4-APA with *E. coli* (Masuo et al., 2016; Konishi et al., 2017). Still, the achieved titers and yields of these aromatic amines are in most cases relatively low and might not be sufficient for an intended industrial use.

The pathway to PAPE runs in analogy to the classical Ehrlich pathway for 2-phenylethanol (2-PE) production from l-phenylalanine in yeasts (Etschmann et al., 2002; Stark et al., 2003; Hua and Xu, 2011; Kim et al., 2014a; Suastegui and Shao, 2016) with the only difference that l-PAPA is the starting amino acid. The Ehrlich pathway to 2-PE consists of a transaminase (aminotransferase) reaction on phenylalanine to deliver phenylpyruvate, which in turn is decarboxylated by a ThDP-dependent keto-acid decarboxylase (KDC) to phenylacetaldehyde. Phenylacetaldehyde can then be reduced to 2-PE by an oxidoreductase (alcohol dehydrogenase, ADH). Alternatively, phenylacetaldehyde can be oxidized to phenylacetate by an aldehyde dehydrogenase (Hazelwood et al., 2008). Whereas, the conversion of l-phenylalanine to 2-PE is a biotransformation from a more expensive starting material, a biosynthesis of 2-PE (or PAPE) would start from an inexpensive carbon source as glucose and include the aromatic biosynthesis pathway to phenylpyruvate/l-phenylalanine (APP/L-PAPA) as well. By establishing these necessary enzyme activities in recombinant *E. coli* cells, Takaya and coworkers reported titers for PAPE and 4-APA titers of about 0.24 and 0.19 g l^−1^ from glucose, respectively, with their genetically modified strains (Masuo et al., 2016).

In the present study, we followed this idea for *de-novo* PAPE/4-APA biosynthesis pathway in *E. coli*. The overall pathway contains an upstream pathway module from glucose to chorismate (naturally occurring in *E. coli*) and a downstream pathway module from chorismate to PAPE or 4-APA via *p-*amino-phenylpyruvate (APP) (see Figure 1). We also took care of improved provision of possibly limiting intermediates. Thereby we were able to improve the PAPE and 4-APA titers.

## Materials and Methods

### Bacterial Strains

All *E. coli* K-12 strains and plasmids employed in this study are listed in Table S1. All used primers are listed in Table S2. *E. coli* DH5α was used for cloning experiments and *E. coli* LJ110 (W3110) was used as wild-type strain.

### Bacterial Culture Medium, Enzymes, and Reagents

Lysogeny broth (LB, 10 g l^−1^ tryptone, 5 g l^−1^ yeast extract, and 10 g l^−1^ NaCl) was used as complex media (Sambrook et al., 2001). The AA production was performed in minimal medium (Gerhardt et al., 1984) which contained 3 g l^−1^ KH_2_PO_4_, 12 g l^−1^ K_2_HPO_4_, 5 g l^−1^ (NH4)_2_SO_4_, 0.3 g l^−1^ MgSO_4_·7H_2_O, 0.1 g l^−1^ NaCl, 0.1125 g l^−1^ Fe(II)SO_4_·7H_2_O/Na citrate 15 ml (from the solution of 7.5 g l^−1^ FeSO_4_ and 100 g l^−1^ sodium citrate), 0.015 g l^−1^ CaCl_2_·2H_2_O, 7.5 μg l^−1^ thiamine, 0.02 mg ml^−1^ l-phenylalanine (l-Phe), 0.02 mg ml^−1^ l-tyrosine (l-Tyr), 3 g l^−1^ aspartic acid (if required) and 4.5 g l^−1^ glucose. The shake flask cultivation was carried out in 250 mL Erlenmeyer flasks containing 20 ml of minimal medium. The cultures were grown at 37°C on an orbital shaker with a stirring speed of 150 rpm. The gene expressions were induced with isopropyl β-D-thiogalactopyranoside (IPTG, the final concentration was 0.5 mM) at an OD_600_ of 0.6. If required, antibiotics were added at appropriate concentrations of kanamycin 50 μg ml^−1^ and ampicillin 100 μg ml^−1^. *para*-amino-phenylethanol (PAPE) was purchased from Sigma-Aldrich Chemie GmbH (Munich, Germany), *para*-amino phenylacetic acid (4-APA) from Merck KGaA (Darmstadt, Germany), IPTG was from PEQLAB Biotechnologie GmbH (Erlangen, Germany) and all other chemical were either from Carl Roth GmbH (Karlsruhe, Germany), Fluka (Taufkirchen, Germany), or Merck KGaA (Darmstadt, Germany) and were of the highest available purity. All restriction enzymes and T4 DNA ligase were purchased from New England Biolabs (Ipswich, USA). KOD Hot Start DNA Polymerase for gene amplification was from Novagen, Merck KGaA (Darmstadt, Germany), Taq DNA polymerase used for screening-PCR was from Genaxxon Bioscience GmbH (Ulm, Germany). Plasmid preparation and gel extraction kits were obtained from Macherey-Nagel (Düren, Germany) and DNA sequencing services were provided by GATC Biotech GmbH (Konstanz, Germany).

### Construction of the Plasmids for PAPE or 4-APA Biosynthesis Pathway

To enable the PAPE or 4-APA biosynthesis we constructed the plasmid pC53BCA, pC53BCAY, pC53BCAHII, or pC53BCAF, respectively. These plasmids are based on pC53BC, which already carries the genes *pabAB*, from *C. glutamicum* and *papBC* from *Streptomyces venezuelae* (Mohammadi Nargesi et al., 2018). The ThDP-dependent ketoacid decarboxylase gene *aro*10 was amplified from genomic DNA of *Saccharomyces cerevisiae* W3118 (Vandenhazel et al., 1992) by using the primer pair *aro10*-Fw/Rw (Table S2). The *aro*10 carrying PCR product was first introduced by blunt-end ligation into the *Eco*RV-restricted cloning vector pBluescript KS to generate pBSK*-aro10* and sequence-verified before continuing with the construction process. Then the *aro*10 containing DNA fragment was isolated by *Bam*HI-*Xba*I restriction which was ligated to a *Bam*HI/*Xba*I restricted plasmid DNA of pJF119EH or pC53BC subsequently to yield pJFA10 and pC53BCA, respectively. Genes encoding alcohol dehydrogenases (I and II) *ADH1* and *ADH2* (De Smidt et al., 2008) from *Saccharomyces cerevisiae* W3118, and genes for aldehyde reductase (*yahK*) and phenylacetaldehyde dehydrogenase (*feaB*) from *Escherichia coli* BW25113 were PCR amplified from genomic yeast or *E. coli* DNA, respectively. The following primer pairs were used: *ADHI*-Fw/-Rw, *ADHII*-Fw/-Rw, *yahK*-Fw/-Rw and *feaB*-Fw/-Rw (Table S2). The resulting PCR products of *ADH1, ADH2, yahK* or *feaB* were each ligated blunt end first into the *Eco*RV restricted pBluescript KS cloning vector to generate pBSK-*ADH*I, pBSK-*ADHII*, pBSK-*yahK*, or pBSK-*feaB*, respectively. The correct sequences were verified by sequencing (GATC, Konstanz, Germany). The fragments containing *ADH1, ADH2, yahK*, or *feaB* genes were double-digested with *Sph*I/*Sbf* I restricted and ligated into a *SphI/SbfI* digested pC53BCA vector to generate pC53BCAHI, pC53BCAHII, pC53BCAY, or pC53BCAF, respectively. The digested fragment containing *feaB* was also ligated to *Sph*I/*Sbf* I restricted pJFA10 expression plasmid to generate pJFA10F. Additionally, *yahK* or *feaB* fragments were digested by *Sma*I/*Kpn*I and then ligated into *Sma*I/*Kpn*I restricted pJNT*aroFBL*, yielding pJNT*aroFBL*-*yahK* or pJNT*aroFBL*-*feaB*, respectively.

### Construction of *aspC, tyrB*, and *tyrR* Deletion Mutants

For chromosomal gene disruption the one-step recombineering inactivation method was used (Datsenko and Wanner, 2000). For PCR amplification of the FRT-flanked chloramphenicol resistance cassette for the *aspC* deletion the primer pair Del-*aspC*-fw/-rev was used. For the deletion of *tyrB*, the primer pair Del-*tyrB*-fw/-rev was used with pCAS30-FRT-*cat*-FRT as template for the chloramphenicol resistance cassette (Tables S1, S2) (Vallon et al., 2008). The obtained DNA amplificates were consecutively transformed into *E. coli* FUS4 (Gottlieb et al., 2014) carrying the λ-Red recombinase expression vector pKD46 (Datsenko and Wanner, 2000). Knock-in mutant colonies were selected on LB agar plates with chloramphenicol (Cm; 30 mg l^−1^) and were tested by colony PCR for correct location using the control primer Ko-*aspC*-fw/-rev or Ko-*tyrB*-fw/-rev (Table S2). After each successful integration the chloramphenicol resistance gene *cat* was removed by using the plasmid pCP20 as previously described (Cherepanov and Wackernagel, 1995). The *tyrR* deletion was performed as described previously (Mohammadi Nargesi et al., 2018). The chromosomal gene deletions were verified by PCR.

### Verification of Aro10 Specificity Toward APP via Whole Cell Biotransformation

To verify the substrate specificity of Aro10 toward *para*-amino-phenylpyruvate (APP), a resting whole cell biotransformation approach was applied. Bio-L-PAPA (wherein “Bio” refers to microbial production of aromatic monomers) was produced from glucose as described previously (Mohammadi Nargesi et al., 2018). An overnight LB culture of *E. coli* LJ110 with pJFA10 or pJFA10F was used to inoculate 50 ml LB media to an OD_600_ of 0.1 in a 500 ml Erlenmeyer flask and was incubated by shaking at 30°C and 150 rpm. At an OD_600_ of about 0.6, IPTG was added to the culture (final concentration 0.5 mM). After 6 h incubation (OD_600_ ~5) the cells were collected by centrifugation (6000 g and 10 min), washed twice with potassium phosphate buffer (200 mM, pH~7.2) and resuspended in a reaction mixture (10 mL) containing ~1 g l^−1^ Bio-L-PAPA, 200 mM potassium phosphate buffer (pH ~ 7.2) and 0.25 mM thiamine diphosphate (ThDP) to start the biotransformation with an OD_600_ ~ 16–18. Resting cells were incubated in a rotary shaking incubator at 110 rpm at 30°C. l-PAPA consumption and PAPE or 4-APA production were determined by taking samples at 2 h intervals over a period of 8 h. The direct product of Aro10 action, *p*-aminophenylacetaldehyde (APAAL), could not be measured.

### Shake Flask Batch and Fed-Batch Cultivation for PAPE or 4-APA Production With *E. coli*

An overnight culture growing in minimal medium was used to inoculate 20 ml of minimal medium in a 250 ml Erlenmeyer flask. The medium was supplemented with 0.02 mg ml^−1^ l-Phe, l-Tyr, 3 mg ml^−1^ l-aspartic acid (if applicable) and 4.5 g l^−1^ glucose. The shake flasks were cultivated at 37°C with 150 rpm. After 6 h (batch cultivation) or 24 h (fed-batch cultivation), IPTG was added to a final concentration of 0.5 mM and the temperature was reduced to 30°C. In order to start the fed-batch cultivation ~ 4.5 g l^−1^ glucose was fed to the culture in intervals of 12 h. In order to increase the biomass production, additional pulses of 0.01 mg ml^−1^ l-Phe and l-Tyr were given concomitant with IPTG addition. During the cultivation 5 g l^−1^, sodium bicarbonate was added to adjust the pH and 4 g l^−1^ ammonia sulfate was given as an ammonia source. Samples were taken every 12 h. A portion of the samples was used for optical cell density measurement, and the rest were centrifuged at 22,000 g for 10 min. The supernatants were stored at −28°C until further analysis. All the experiments in this report were conducted in three independent replicates.

### PAPE and 4-APA Susceptibility Assays

PAPE or 4-APA susceptibility of *E. coli* strains was determined by monitoring cell growth at various PAPE or 4-APA concentrations by measuring the OD_600nm_ (Cary 50 Bio, Varian). An overnight culture grown in minimal media with 4.5 g l^−1^ glucose was used to inoculate the minimal media with an initial OD600 of 0.1. Cell growth was monitored for 48 h.

### Analytical Methods

To analyze PAPE and 4-APA amounts, culture samples were centrifuged at 22,000 g for 10 min. The supernatants were transferred to new tubes and were frozen at −28°C until further use. The PAPE and 4-APA concentrations were measured by high-pressure liquid chromatography (HPLC; Agilent Technologies Series 1200 system, USA) using a symmetry C18 silica column (Prontosil, 250 × 4 mm, CS Chromatography Service GmbH, Langerwehe, Germany) with a pre-column. 40 mM Na_2_SO_4_ (pH adjusted ~2.7 with methanesulfonic acid) were used as the mobile phase with a flow rate of 1 ml min^−1^ at 40°C. Absorption was detected at 210 nm with a DAD detector (Agilent Technologies Series 1260 Infinity DAD system). For LC-MS analysis of PAPE and 4-APA, the same HPLC method was used as described above, except that the mobile phase was replaced by 0.1% formic acid solution. The mass was determined using an Agilent 1260 system with 1260 Infinity DAD detector and an Agilent 6130 mass spectrometer (Agilent Technologies Waldbronn, Germany). Glucose concentration was monitored by using Glucose Medi-Test stripes (Macherey Nagel, Düren, Germany) and by HPLC on an Organic acid column (300 × 8 mm, CS Chromatography Service GmbH, Langerwehe, Germany) at 40°C. 5 mM H_2_SO_4_ was used as mobile phase with a flow rate of 0.6 ml min^−1^. Absorption was detected by the refractive index (RI) detector. The compounds were quantified using standard curves of the respective commercial chemicals.

## Results

### Susceptibility of *E. coli* Cells to PAPE and 4-APA

As it is known that 2-phenylethanol (2-PE), a structural analog of PAPE, is toxic to *E. coli* at doses above 1–1.5 g l^−1^ (Masker and Eberle, 1972; Lucchini et al., 1993; Kang et al., 2012), we tested the growth behavior of *E. coli* toward the addition of PAPE or 4-APA. The PAPE or 4-APA susceptibility was assayed by monitoring the biomass formation of *E. coli* wild-type strain LJ110 in minimal medium with varying concentrations of PAPE or 4-APA. At concentrations up to 30 mM of PAPE or 4-APA, only little effects on the growth of *E. coli* were observed (Figure S1). Cells were still able to grow in the presence of 40 mM PAPE or 4-APA, but the growth rate (μ) was decreased to 0.27 h^−1^ (PAPE) or 0.38 h^−1^ (4-APA) compared to the absence (μ~0.67 h^−1^). A complete growth inhibition was observed at a PAPE or 4-APA concentration of 50 mM (Figure S1). From this observation we reasoned that PAPE and 4-APA are compounds to be produced with recombinant *E. coli* strains without extreme toxicity to the producer cells.

### Construction of a Recombinant Route to PAPE and 4-APA in *E. coli*

In order to enable the production of the aromatic amines PAPE and/or 4-APA in *E. coli* a recombinant route has to be established. The key metabolite chorismate needs to be converted to *p*-amino-phenylpyruvate (APP) via 4-amino-4-deoxy-chorismate (ADC), which is also the precursor of PABA (Figure 1). For this purpose, we used the plasmid pC53BC, which carries the fused gene *pabAB* (ADC synthase) from *Corynebacterium glutamicum* (Wubbolts et al., 2005; Kozak, 2006; Stolz et al., 2007)*, papB* (ADC mutase) and *papC* (*p-*amino-4-deoxyprephenate dehydrogenase) from *Streptomyces venezuelae* (Brown et al., 1996; He et al., 2001; Mohammadi Nargesi et al., 2018) (Figures 1, S3). We showed in a recent study the biosynthesis of l-PAPA in recombinant *E. coli* strains using pC53BC (Mohammadi Nargesi et al., 2018). In order to form PAPE and 4-APA, a decarboxylation of APP to *p*-amino-phenylacetaldehyde (APAAL) is necessary. In bacteria, fungi and yeasts certain ThDP-dependent enzymes are known to decarboxylate 2-keto-acids like pyruvate or phenylpyruvate to the corresponding aldehydes (Delaplaza et al., 2004; Siegert et al., 2005; Vuralhan et al., 2005; Yep et al., 2006, 2008; Küberl et al., 2011; Masuo et al., 2015). The ThDP-dependent 2-keto-acid decarboxylase Aro10 from *Saccharomyces cerevisiae* has been described (Vuralhan et al., 2005; Kneen et al., 2011) to have a broad substrate specificity and thus might also accept the *p*-amino-substituted APP. Therefore, we cloned the *aro*10 gene from *S. cerevisiae* strain W3118 and expressed it in *E. coli* (Figure S3). If APP would be used as substrate by Aro10, *p-*amino-phenylacetaldehyde (APAAL) and CO_2_ would be formed. Subsequently, APAAL could be further converted either reductively to the alcohol PAPE (Atsumi et al., 2010; Koma et al., 2012; Rodriguez and Atsumi, 2014) or by oxidation to the acid 4-APA (Hanlon et al., 1997) (see Figure 1). An Aro10 enzyme assay with APP, however, was not possible as this compound is not commercially available and should be rather unstable, as previously reported for 4-amino-4-deoxychorismate and 4-amino-4-deoxyprephenate (Teng et al., 1985; Wubbolts et al., 2005; Konishi et al., 2017).

**Figure 2:**
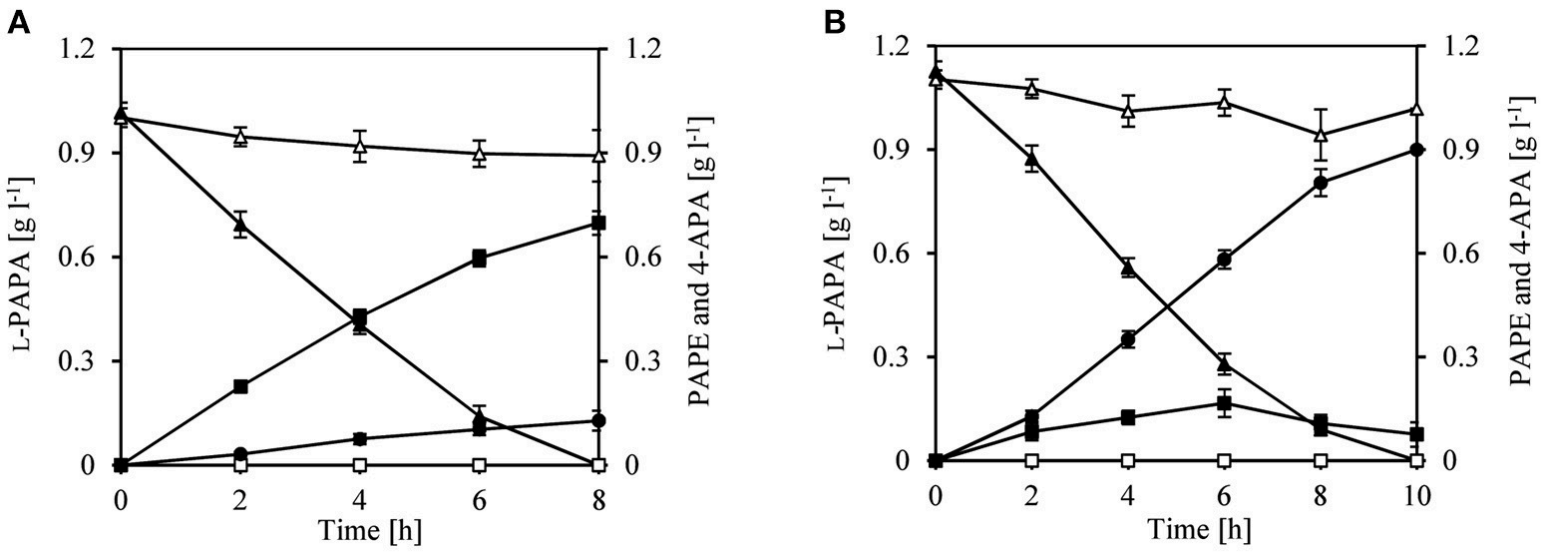
Whole cell biotransformation of *para*-amino-L-phenylalanine (l-PAPA) with resting cells of *E. coli* LJ110/pJFA10 and LJ110/pJFA10F. The Biotransformation was performed at 30°C on a rotary shake with 110 rpm and an initial L-PAPA concentration of 1–1.1 g l^−1^ (triangles). Resting cells of *E. coli* LJ110/pJF119EH (empty symbols), LJ110/pJFA10 (**A**; filled symbols) and LJ110/pJFA10F (**B**; filled symbols) were used. The PAPE formation (squares) and 4-APA (circles) were determined by HPLC. The data represent the mean and standard deviations from measurements of three biological replicates.

We therefore decided to first perform a biotransformation assay with l-PAPA as surrogate substrate; L-PAPA had been previously purified from culture supernatants of a recombinant *E. coli* producer strain (“Bio”-l-PAPA) (Mohammadi Nargesi et al., 2018). We reasoned that l-PAPA can be taken up by *E. coli* cells and can be transaminated intracellularly to APP by the existent aminotransferases (TyrB and/or AspC). For the biotransformation approach, we used *E. coli* LJ110/pJF119EH as control and *E. coli* LJ110/pJFA10 to enable a plasmid borne expression of *aro*10. Furthermore, we used *E. coli* LJ110/pJFA10F to express additionally *feaB* from *E. coli* encoding a phenylacetaldehyde dehydrogenase (Ferrandez et al., 1997; Hanlon et al., 1997; Koma et al., 2012; Zhang et al., 2017). After 8 h of incubation, conversion of l-PAPA neither to PAPE nor to APA could be detected by *E. coli* LJ110/pJF119EH cells (Figure 2). l-PAPA was converted to PAPE by *E. coli* LJ110/pJFA10 with a conversion yield of 0.69 g g l^−1^ PAPE/l-PAPA and a titer of 0.69 ± 0.034 g l^−1^ PAPE (Figure 2A). A conversion yield of 0.80 g g l^−1^ 4-APA/l-PAPA with *E. coli* LJ110/pJFA10F was detected with a titer of 0.89 ± 0.032 g l^−1^ (Figure 2B). Reciprocally, 0.12 ± 0.03 g l^−1^ 4-APA were detected with *E. coli* LJ110/pJFA10 cells and 0.075 ± 0.005 g l^−1^ PAPE in *E. coli* LJ110/pJFA10F as by-products. This indicated that APAAL is diverted to at least two different products (Figure 2).

**Figure 3:**
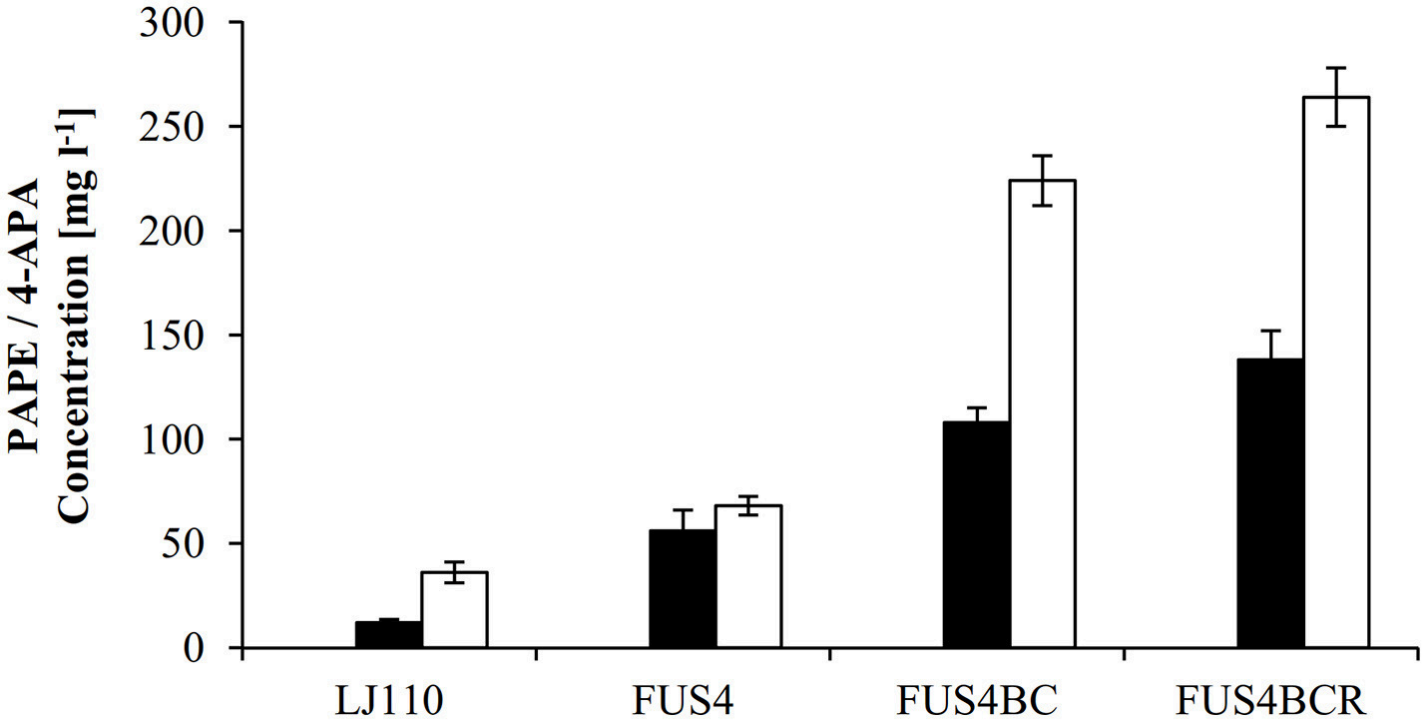
Overview of PAPE or 4-APA production by different *E. coli* strains. The *E. coli* strains LJ110, FUS4, FUS4BC, and FUS4BCR with either pC53BCA for PAPE production (filled columns), or with pC53BCAF for 4-APA production (empty columns) were compared. The PAPE and 4-APA titers were determined by HPLC measurements after 48h batch cultivation in minimal media with 4.5 g l^−1^ glucose. The data represent the mean and standard deviations from measurements of three biological replicates.

Apparently, Aro10 accepted APP as substrate and catalyzed the synthesis of the precursor of PAPE and APA, APAAL. Based upon this finding, we went on to construct the plasmids pC53BCA and pC53BCAF (Figure S3) to allow *in vivo* biosynthesis of either PAPE or APA from glucose as carbon source. Plasmid pC53BCA contains *aro*10, whereas pC53BCAF contains both *aro*10 and *feaB* (see Table S1 and Figure S3). These plasmids were introduced into wild type strain *E. coli* LJ110. When grown in shake flasks with glucose as sole carbon source, the resulting strains *E. coli* LJ110/pC53BCA produced 11 ± 1.5 mg l^−1^ PAPE or *E. coli* LJ110/pC53BCAF produced 36 ± 5 mg l^−1^ 4-APA (Figure 3). While we took this as an initial proof that our strategy worked, it clearly left room for improvement in productivities.

### Elimination of Competing Pathways and Negative Regulation to Enhance Carbon Flux Toward PAPE or 4-APA

To augment PAPE or 4-APA production, we then turned to a more advanced *E. coli* LJ110-derived strain, FUS4 (Gottlieb et al., 2014) which we also had successfully used for L-PAPA formation (Mohammadi Nargesi et al., 2018). This strain carries gene deletions of *pheA* (chorismate mutase/prephenate dehydratase) and *tyrA* (chorismate mutase/prephenate dehydrogenase), as these gene products directly compete for the same precursor, chorismate. Furthermore, an extra copy of *aroFBL* (as a gene cassette) is chromosomally inserted (see Table S1). When grown in shake flasks with glucose as sole C source, the PAPE titer was enhanced to 56 ± 10 mg l^−1^ (Figure 3) with *E. coli* FUS4/pC53BCA. On the other hand, the 4-APA titer was also increased to 68 ± 4.5 mg l^−1^ by *E. coli* FUS4/pC53BCAF.

Next, to reduce the competing carbon flux toward L-PAPA by transamination of APP by TyrB and AspC (Hayashi et al., 1993), the strain FUS4BC was applied. FUS4BC has gene deletions of the aminotransferase genes *tyrB* and *aspC*, which leads to an aspartic acid auxotrophy (see Table S1). FUS4BC cells, when transformed with pC53BCA and pC53BCAF, showed increased PAPE or 4-APA titers of 108 ± 7.5 and 224.6 ± 14.2 mg l^−1^ (Figure 3), respectively. Thus, we concluded that the aminotransferases TyrB and AspC are, indeed, competitors for APP and their removal allows improved formation of both, PAPE or 4-APA.

The transcriptional regulator TyrR (gene *tyrR*) is involved in the tight regulation of genes involved in shikimate pathway (Pittard et al., 2005). We had already seen positive effects on L-PAPA production with *tyrR*-negative strains of *E. coli* (Mohammadi Nargesi et al., 2018). To alleviate TyrR regulation, *tyrR* gene was thus deleted in strain FUS4BC to yield FUS4BCR. After introduction of pC53BCA or pC53BCAF into strain FUS4BCR, we grew the strains in shake flasks with glucose as sole C source. The respective product titers increased further to 138 ± 8 mg l^−1^ PAPE and 264 ± 12 mg l^−1^ 4-APA (Figure 3). This is about 2.5 and 4 times higher than FUS4/pC53BCA or FUS4/pC53BCAF, respectively (Figure 3) and significantly higher than with strain FUS4BC. During PAPE production, 23 ± 7.5 mg l^−1^ 4-APA as by-product was observed in FUS4BCR/pC53BCA. We assume that accumulation of 4-APA by FUS4BCR/pC53BCA is due to an endogenous phenylacetaldehyde dehydrogenase (FeaB, AldB and/or AldH) activity in *E. coli* as described before (Ho and Weiner, 2005; Jo et al., 2008; Koma et al., 2012; Zhang et al., 2017).

### Effect of Additionally Expressed Dehydrogenase/Reductase Activities on PAPE Synthesis

As described above, Aro10 apparently is able to produce APAAL from APP. This aldehyde then can either be reduced to an alcohol (PAPE) or may be oxidized to 4-APA. In *E. coli*, there have been several reports on various genes which encode real or predicted activities of alcohol dehydrogenases/aldehyde reductases (Atsumi et al., 2008a; Koma et al., 2012; Kunjapur et al., 2014; Rodriguez and Atsumi, 2014) or of phenylacetaldehyde dehydrogenase (Hanlon et al., 1997; Koma et al., 2012; Zhang et al., 2017). As we looked for genes which encode aldehyde reductases or alcohol dehydrogenase which might facilitate the PAPE production in our *E. coli* strains, we decided to clone heterologous genes from the yeast *S. cerevisiae* (alcohol dehydrogenase genes *ADH1* and *ADH2*) as well as the endogenous *E. coli yahK* gene, which had been proposed to be most efficient for the production of aromatic alcohols (Koma et al., 2012). The three genes were cloned separately into plasmid pC53BCA to yield the new plasmids pC53BCAHII, pC53BCAHI, and pC53BCAY (Figure S3). Each plasmid was then introduced into our best producer stain so far, FUS4BCR (Table S1, Figure S3). The cell growth and the PAPE formation were observed for 48 h in shake flask batch cultures with glucose as sole C source.

First, to our surprise, FUS4BCR/pC53BCAHII did not produce PAPE at all; rather 4-APA was detected at 85 mg l^−1^ (Table 1). This finding could be rationalized if the recombinant ADHII enzyme in *E. coli* either does not reduce APAAL to PAPE, or even catalyzes the re-oxidation of PAPE thereby counteracting the action of endogenous *E. coli* aldehyde reductases.

**Table 1:** PAPE and 4-APA production by *E. coli* FUS4BCR strains harboring different combinations of plasmids.

**Plasmids**	**Titer (mg l**^****−1****^**)**		**Yield (%, g g** ^****−1****^**)**	
	**PAPE**	**4-APA**	**PAPE**	**4-APA**
pJF119EH	n.d.	n.d.	n.a.	n.a.
pC53BCAHI	34 ± 5	15 ± 4	0.7	0.3
pC53BCAHII	n.d.	85 ± 5	n.a.	1.8
pC53BCAY	159 ± 14	11 ± 5	3.5	0.2
pC53BCAF	n.d.	264 ± 12	n.a.	5.8
pC53BCAY/pJNT*aroFBL*	263 ± 25	26 ± 7	5.8	n.a.
pC53BCAF/pJNT*aroFBL*	n.d.	307 ± 12	n.a.	6.8
pC53BCAY/pJNT*aroFBL*-*yahK*	526 ± 25	n.d.	11	n.a.
pC53BCAF/pJNT*aroFBL*-*feaB*	n.d.	458 ± 14	n.a.	10

*The strains were cultivated for 48 h in minimal media with 4.5 g l^−1^ of glucose. The glucose was fully consumed and the formation of PAPE and 4-APA was detected by HPLC. The data represent the means and standard deviations from measurements of three biological replicates. n.d., not detectable; n.a., not applicable*.

The expression of *ADH1* (plasmid pC53BCAHI) resulted in a titer of about 40 mg l^−1^ while the overexpression of the plasmid-borne *yahK* led to an increased PAPE titer of 159 mg l^−1^ (Table 1). Thus, from the tested genes, *yahK* overexpression gave best results for PAPE production.

### Plasmid-Borne Overexpression of Genes to Further Enhance PAPE or 4-APA Production

One common strategy to enhance the flux through the shikimate pathway is increasing the activity of major rate-limiting steps in the pathway (Báez-Viveros et al., 2007; Sprenger, 2007a; Gosset, 2009; Chávez-Béjar et al., 2013; Gottlieb et al., 2014). Hence to improve the flux toward chorismate as the main precursor for APP biosynthesis, we turned to a two-plasmid system with the additional vector pJNT-*aroFBL* (Mohammadi Nargesi et al., 2018) which confers kanamycin resistance. This vector is compatible with the pC53 plasmid series and was introduced into FUS4BCR/pC53BCAY and FUS4/pC53BCAF. In shake flask batch cultivations, the PAPE titer of FUS4BCR/pC53BCAY/pJNT-*aroFBL* increased to 263 ± 15 mg l^−1^ PAPE (Table 1), which was ~2-fold higher compared to the FUS4BCR/pC53BCAY (Table 1). Within 48 h, FUS4BCR/pC53BCAF/pJNT-*aroFBL* consumed 4.5 g l^−1^ glucose completely and produced 307 ± 12 mg l^−1^ 4-APA, which is about 1.5-fold more than the concentration of 4-APA observed with the one plasmid strain FUS4BCR/pC53BCAF.

To see whether an additional copy of either *yahK* or *feaB* genes from *E. coli* would lead to a further increase of products, they were cloned into pJNT-*aroFBL* generating pJNT-*aroFBL*-*yahK* and pJNT-*aroFBL*-*feaB*, respectively (Figure S3). These additional gene overexpressions led to a final titer of PAPE (526 ± 25 mg l^−1^) in the case of *yahK* and 4-APA (458 ± 14.5 mg l^−1^) in the case of *feaB*. In both strains no reciprocal by-product (4-APA and PAPE, respectively) was detectable (Table 1). Therefore, we decided to use strain FUS4BCR/pC53BCAY/pJNT-*aroFBL-yahK* and FUS4BCR/pC53BCAF/pJNT-*aroFBL-feaB* for fed-batch cultivations for PAPE or 4-APA, respectively.

### Fed-Batch Cultivations for PAPE and 4-APA Production

**Figure 4:**
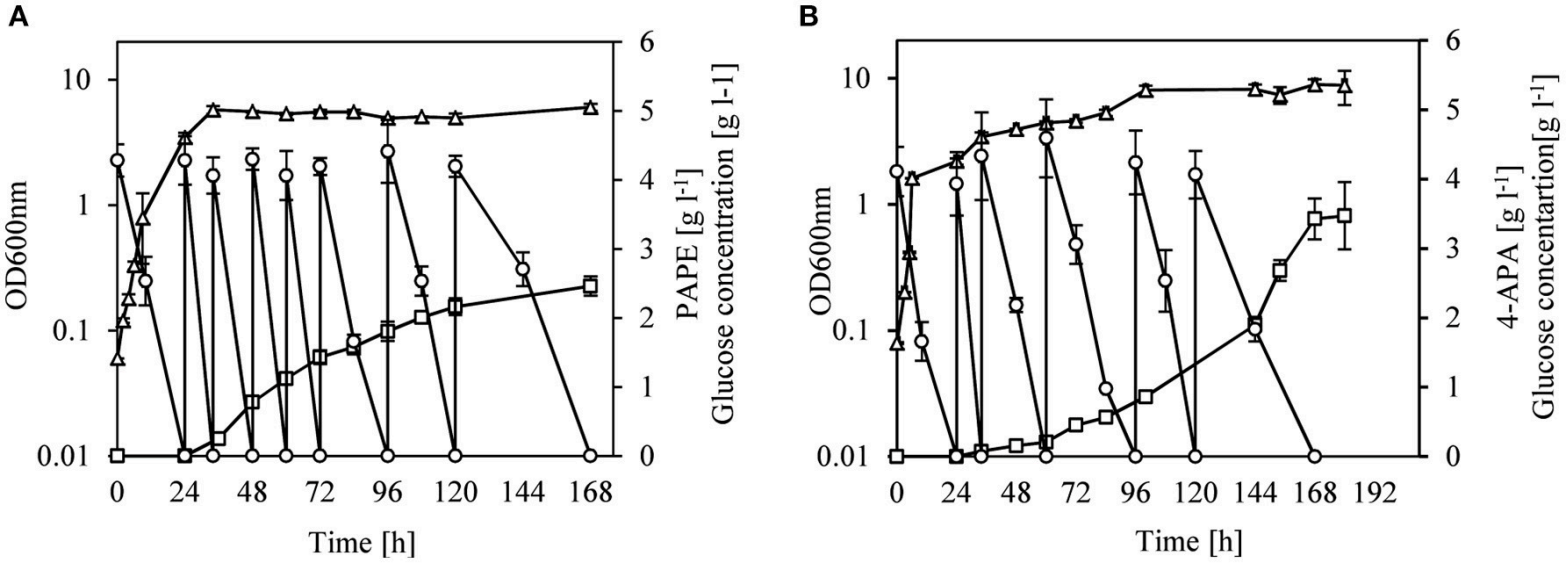
Fed-batch cultivation of *E. coli* FUS4BCR/pC53BCAY/pJNT*aroFBL-yahK* **(A)** or *E. coli* FUS4BCR/pC53BCAF/pJNT*aroFBL-feaB* **(B)** for PAPE or 4-APA production. Cell growth (empty triangles) was determined by measuring OD_600_, glucose concentration (empty circles) and the total PAPE or 4-APA production (empty squares) in the minimal media were determined by using HPLC. The cells were cultivated in 20 ml minimal media for 168 h. The data represent the mean and standard deviations from measurements of three biological replicates.

We changed the cultivation conditions to fed-batch to see whether product titers of PAPE and 4-APA could be further improved. After 168 h of cultivation of strain FUS4BCR/pC53BCAY/pJNT-*aroFBL-yahK*, 33.3 g l^−1^ glucose were consumed and a titer of 2.5 ± 0.15 g l^−1^ PAPE with a yield of 0.08 PAPE/g of glucose (11% C mol PAPE mol^−1^ glucose) was reached (Figure 4A). FUS4BCR/pC53BCAF/pJNT-*aroFBL-feaB* gave a final product concentration of 3.4 ± 0.3 g l^−1^ 4-APA from 25.3 g l^−1^ glucose with a yield of 0.14 4-APA/g of glucose (17% C mol 4-APA mol^−1^ glucose) after 168 h (Figure 4B). To the best of our knowledge these are the highest reported PAPE and 4-APA titers with recombinant *E. coli* strains.

## Discussion

In this study we established a *de novo* biosynthesis pathway for two aromatic amine products, PAPE and 4-APA in *E. coli* which share a common precursor, APAAL. We could demonstrate that *E. coli* can efficiently produce PAPE and 4-APA for the first time in gram scale from glucose. Recently, Takaya and coworkers have also studied the microbial biosynthesis of PAPE and 4-APA with recombinant *E. coli* strains (Masuo et al., 2016). However, to reach the high titer of 2.8 g l^−1^ PAPE and 2.7 g l^−1^ 4-APA, these authors used whole cell biotransformations using the expensive l-PAPA as substrate with *E. coli* cells overexpressing the *aro*10 gene from yeast and relying on the endogenous aminotransferase activities of *E. coli* (Masuo et al., 2016).

From the results obtained by the Takaya group and the present report with biotransformation with externally added l-PAPA as substrate it becomes apparent that the gene product of the yeast *aro*10 gene encodes a decarboxylase with a broader substrate range than reported before (Vuralhan et al., 2005; Kneen et al., 2011). We should emphasize however that we could not detect formation of the aldehyde APAAL; rather, we detected the follow-up products, either the reduced PAPE or the oxidized 4-APA. The approach of Takaya and coworkers differs from our report as the former group applied a synthetic gene cluster of *papABC* from *Pseudomonas fluorescens* for *para*-amino phenylpyruvate (APP) production in *E. coli* (Masuo et al., 2016). In our study, a combination of the *pabAB* fusion gene from *C. glutamicum* and of the codon-optimized genes of *papB* and *papC* from *S. venezuelae* was used to allow a conversion of chorismate to APP (Mohammadi Nargesi et al., 2018). We combined the APP biosynthesis pathway with the yeast Ehrlich pathway in *E. coli* by recruiting Aro10 to enable PAPE and 4-APA production (Figure 1) (Vuralhan et al., 2003, 2005; Vogt and Gerulis, 2005; Atsumi et al., 2008b; Kneen et al., 2011; Machas et al., 2017; Mohammadi Nargesi et al., 2018). Already the wild type strain *E. coli* LJ110 harboring pC53BCA or pC53BCAF was able to produce 11 ± 1.5 mg l^−1^ PAPE or 36 ± 5 mg l^−1^ 4-APA; the correct mass of these products was confirmed by mass spectrometry (Figure S2). This result indicated that the *de-novo* PAPE or 4-APA biosynthesis pathway was successfully established and that the endogenous aldehyde reductases/dehydrogenases of *E. coli* (Atsumi et al., 2008b, 2010; Koma et al., 2012; Kunjapur et al., 2014; Rodriguez and Atsumi, 2014) are able to convert APAAL to PAPE or 4-APA, respectively.

In order to improve the flux toward the products, we decided to use more advanced *E. coli* strains. *E. coli* strain FUS4 (Gottlieb et al., 2014) carries gene deletions (*pheA-tyrA*) which remove the activities of the bifunctional enzymes and thus allow increased chorismate flux to the desired products as the formation of l-Tyr and l-Phe is disabled (Backman et al., 1990; Rüffer et al., 2004; Sprenger, 2007a,b; Sun et al., 2011; Gottlieb et al., 2014; Weiner et al., 2014). As a certain disadvantage for growth in minimal media, however, supplementation of both aromatic amino acids is necessary. Strain FUS4-when transformed with plasmids pC53BCA or pC53BCAF, respectively—yielded increased titers of PAPE or 4-APA compared to the wild type host strain. The PAPE titer was improved five times to 56 ± 10 mg l^−1^ while the 4-APA titer was almost doubled to 68 ± 4.5 mg l ^−1^ (Figure 3). Thus, the removal of competing pathways resulted in an improved formation of PAPE and/or 4-APA.

In *E. coli*, several aminotransferases are known to catalyze terminal steps in aromatic amino acid biosynthesis (Fotheringham et al., 1986; Inoue et al., 1988; Hayashi et al., 1993; Pittard, 1996; Marienhagen et al., 2005). For the transamination of the aromatic amino acids l-phenylalanine and l-tyrosine, four aminotransferases (AspC, TyrB, IlvE, and AvtA) are involved in *E. coli* (Pittard, 1996; Rodriguez et al., 2014; Li et al., 2016). Previous studies had already shown that the deletions of genes *aspC* and *tyrB* led to an aspartic acid auxotrophy but also to an increased precursor supply of aromatic ketoacids like phenylpyruvate or 4-hydroxyphenylpyruvate (Liu et al., 2014, 2015; Pugh et al., 2014; Li et al., 2016). In good agreement with these former studies, the use of *E. coli* FUS4BC which lacks *aspC* and *tyrB*, led to an almost doubling of the titer for PAPE to 108 ± 7.5 mg l^−1^ and the 4-APA titer of 224.6 ± 14.2 mg l^−1^ was 3-fold higher (Figure 3).

Furthermore, the transcriptional repressor *tyrR* was deleted as its inactivation is known to enhance the expression of aromatic biosynthesis genes, like *aroG, tyrB, aroP, tyrA*, and *aroL* (Pittard, 1996; Bongaerts et al., 2001; Pittard et al., 2005; Salgado et al., 2006). The beneficial effect of a *tyrR* deletion had been already shown for the biosynthesis of aromatic amino acids like l-Tyr (Lutke-Eversloh and Stephanopoulos, 2007), l-Phe (Doroshenko et al., 2015), l-DOPA (Munoz et al., 2011; Das et al., 2018), and recently l-PAPA (Mohammadi Nargesi et al., 2018). The cultivation of FUS4BCR/pC53BCA or/pC53BCAF increased the PAPE or 4-APA titer up to 27 or 17% than FUS4BC/pC53BCA or/pC53BCAF, respectively (Figure 3).

The reduction or the oxidation of APAAL to PAPE and 4-APA in *E. coli* is accomplished by endogenous aldehyde reductases encoded by the *yqhD, yjgB, yahK*, or other genes (in total 13 known endogenous aldehyde reductases) (Atsumi et al., 2008a; Koma et al., 2012; Rodriguez and Atsumi, 2014) or phenylacetaldehyde dehydrogenase encoded by *feaB* in *E. coli* (Hanlon et al., 1997), respectively. Although *E. coli* FUS4BCR/pC53BCA produced PAPE already with a reasonable titer, a formation of the by-product 4-APA with ~23 ± 7.5 mg l^−1^ was detected. This indicated that the endogenous dehydrogenase gene (*feaB*) in the genome of *E. coli*, enables the conversion of APAAL to 4-APA, which limits the PAPE production (Masuo et al., 2016; Machas et al., 2017). To avoid an unwanted 4-APA accumulation we tested three genes *yahK, ADH1*, and *ADH2* for their potential roles in accelerating PAPE formation as the role of these genes had been discussed before by others (Dickinson et al., 2003; Koma et al., 2012; Kim et al., 2014a,b). Unexpectedly however, introduction of pC53BCAHII into strain *E. coli* FUS4BCR resulted in a complete loss of PAPE production (Table 1). This could be explained by a preference of the recombinant yeast ADHII enzyme to catalyze the oxidation of alcohols instead of a reduction of aldehydes (Thomson et al., 2005; De Smidt et al., 2008; Kang et al., 2012). When the gene for ADHI from yeast was introduced on pC53BCAHI plasmid into strain FUS4BCR this led to lower level of PAPE than in the strain without an extra alcohol dehydrogenase FUS4BCR/pC53BCA, e.g., 34.3 ± 5.2 mg l^−1^ (see Figure 3 and Table 1). Also for ADHI enzyme reports pointed to its ability to oxidize primary alcohols at high concentrations (Schöpp and Aurich, 1976; De Smidt et al., 2008; Atsumi et al., 2010). So in comparison of the three alcohol dehydrogenases/aldehyde reductases which we tested, the cloned endogenous aldehyde reductase encoded by *yahK* (Koma et al., 2012; Rodriguez and Atsumi, 2014) on pC53BCAY led to increased PAPE production in *E. coli* FUS4BCR. Simultaneously this led to a reduction of the by-product 4-APA to 11 ± 5 mg l^−1^ (Table 1). To ensure a sufficient supply of chorismate for the biosynthesis of PAPE or 4-APA, an increased carbon flux through the shikimate pathway is needed. This can be achieved by deregulation of expression of *aroF* (DAHP synthase) and relieving limiting enzymatic reactions of DHQ synthase (encoded by *aroB*) and shikimate kinase II (encoded by *aroL*) (Dell and Frost, 1993; Oldiges et al., 2004; Báez-Viveros et al., 2007; Sprenger, 2007b). Therefore, we used the plasmid pJNT-*aroFBL* to overexpress the genes *aroF, aroB*, and *aroL* (Mohammadi Nargesi et al., 2018). Indeed, the two plasmid combination of pJNT*aroFBL* with pC53BCAY or pC53BCAF in FUS4BCR strains, resulted in an increased carbon flux to chorismate and eventually to the desired products. The co-expression of *aroFBL* led to about 1.5-fold increase of PAPE or 4-APA titer in FUS4BCR pC53BCAY/pJNT*aroFBL* (263 ± 15 mg l^−1^) or FUS4BCR pC53BCAF/pJNT-*aroFBL* (307 ± 12 mg l^−1^). The yield was increased about 40% from 3.5% PAPE/glucose (g g^−1^) (Table 1) to 5.8% PAPE/glucose (g g^−1^) and a 17% increase in yield was observed for 4-APA (Table 1). This beneficial effect of an increased flux through the shikimate pathway for PAPE and 4-APA formation was also shown for other compounds derived from the key metabolite chorismate (Sprenger, 2007b; Kang et al., 2012; Rodrigues et al., 2013; Yao et al., 2013; Gottlieb et al., 2014; Lin et al., 2014; Weiner et al., 2014; Mohammadi Nargesi et al., 2018).

As small amounts of PAPE or 4-APA were still detectable as byproducts during the production of 4-APA or PAPE (Table 1), respectively, we decided to integrate an additional gene copy of genes *yahK* or *feaB* or on the vector pJNT-*aroFBL*. Compared to the previous strains, the additional gene copies of *yahK* or *feaB* caused a 90% and 47% enlargement of the PAPE and 4-APA titers (Table 1). The unwanted side-reactions for PAPE or 4-APA (oxidation or reduction of APAAL, respectively) could be avoided without inactivation of dehydrogenase (*feaB*) or oxidoreductase (*yahK, yqhD, yjgB*, and the other 10 genes), respectively (Rodriguez and Atsumi, 2014; Machas et al., 2017).

Finally, to increase the PAPE and 4-APA titers, the cultivation condition was changed to a fed-batch cultivation in shake flask. A high titer of 2.5 ± 0.15 g l^−1^ PAPE or 3.4 ± 0.3 g l^−1^ 4-APA could be reached which corresponds to a yield of 11 or 17% carbon mol mol^−1^ with glucose after 168 h (Figure 4). This titer is higher than the previously described titers of PAPE (0.24 g l^−1^) and 4-APA (0.19 g l^−1^) in minimal media with 20 g l^−1^ glucose (supplemented with tryptone and yeast extract) from the Takaya group (Masuo et al., 2016; Konishi et al., 2017). Even though we reached the gram scale in shake flask the cultivation was not optimal in term of oxygen supply and pH stability. A cultivation in a pH- and O_2_-controlled bioreactor could be advantageous and will be analyzed in future studies. Furthermore, as we observed a negative effect of PAPE and 4-APA on the growth of *E. coli* at higher concentration (Figure S1), a deletion of the global stress regulator *rpoS* may be beneficial for the aromatic amine production as it was seen for the production of the diamine putrescine (Qian et al., 2009).

To further augment the PAPE or 4-APA production performance we have to consider to improve the E4P and PEP precursor supply for future strain developments (Patnaik et al., 1995; Bongaerts et al., 2001; Chandran et al., 2003; Sprenger, 2007b; Gosset, 2009; Rodriguez et al., 2014). It is very likely that a positive effect will also be observed for PAPE and 4-APA production as we detected it for l-phenylalanine and l-PAPA production (Gottlieb et al., 2014; Mohammadi Nargesi et al., 2018; Trondle et al., 2018). In addition an even more improved flux through the shikimate pathway may increase the productivity as it was demonstrated that an increased flux through the l-lysine pathway augmented the biosynthesis of the diamine cadaverine (Qian et al., 2011). Although we already minimized the byproduct formation by overexpression of *feaB* or *yahK*, we have to consider also to eliminate the genes to suppress phenylacetic acid/4-hydroxyphenylacetic acid pathway in a further improved process as it was done previously (Satoh et al., 2012; Bai et al., 2014; Machas et al., 2017; Xue et al., 2017; Liu et al., 2018).

In conclusion, our study demonstrated that *E. coli* is a suitable chassis strain for both, PAPE and 4-APA production. By a combination of improved flux, avoidance of by-product formation and a change in the cultivation condition a gram scale production of PAPE and 4-APA was achieved.

## Availability of Data and Materials

The dataset(s) supporting the conclusions of this article are all included within the article and additional files.

## Author Contributions

BM performed the experiments. GS and J-WY provided guidance for the experimental setups. BM, GS, and J-WY wrote the final manuscript. All authors approved the final version of the manuscript.

### Conflict of Interest Statement

The authors declare that the research was conducted in the absence of any commercial or financial relationships that could be construed as a potential conflict of interest.
