# Supplementary material for: Metabolic Engineering of Escherichia coli for para-Amino-Phenylethanol and para-Amino-Phenylacetic Acid Biosynthesis
Source: Front Bioeng Biotechnol. 2019 Jan 4;6:201. doi: 10.3389/fbioe.2018.00201 (PMC6328984; doi:10.3389/fbioe.2018.00201)
Supplement: Supplementary file 1 [file Table_1.docx]

Supplementary Material

Metabolic Engineering of *Escherichia coli* for *para*-Amino-Phenylethanol and *para*-Amino-Phenylacetic Acid Biosynthesis

Behrouz Mohammadi Nargesi^1^, Georg A. Sprenger^1^, Jung-Won Youn^1*^

^1^Institute of Microbiology, University of Stuttgart, Stuttgart, Germany

*** Correspondence:**Corresponding Author
jung-won.youn@imb.uni-stuttgart.de

**b**

**a**

|  |  |
| --- | --- |

**Figure A 1.** **Growth experiment of *E. coli* LJ110 in minimal medium with 4.5 g l^-1^ glucose in the presence of different concentrations of PAPE (a) or 4-APA (b)** The growth was determined by measuring the OD at 600 nm with a spectrophotometer (Cary 50 UV-Vis, Varian). The concentrations of PAPE or 4-APA were added to the medium as follows: 0 mM (filled square); 7.5 or 10 mM (filled circle); 15 or 20 mM (filled triangle); 30 mM (empty circle); 40 mM (empty square) and 50 mM (empty triangle).


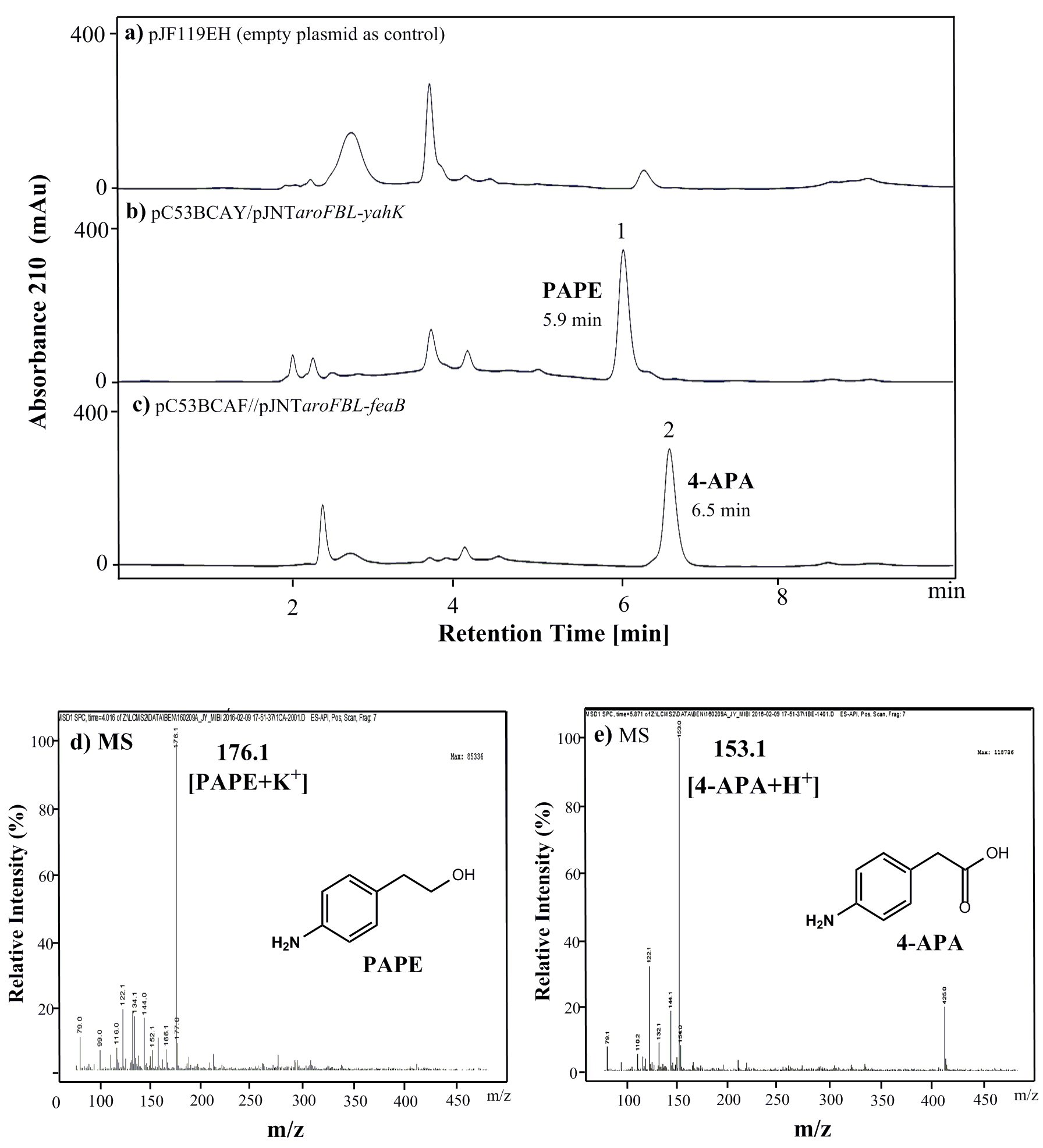


**Figure A 2.** **HPLC Chromatogram (a-c) and mass ion chromatogram (d-e) of PAPE [1, b, d] or 4-APA [2, c, e] produced by *E. coli***. The chromatograms were derived from extract of a culture of *E. coli* FUSBCR harboring empty plasmid pJF119 (a), pC53BCAY/pJNT*aroFBL-yahK* plasmid (b) and pC53BCAF/pJNT*aroFBL-feaB* plasmid (c) grown with glucose as carbon source for 48 h. Culture medium was separated by an HPLC setup as described in material and method, and absorbance was recorded at 210 nm. The peak at 5.9 min [1] (b) represent PAPE and peak at 6.5 min [2, c] represent 4-APA which is not present in *E. coli* FUSBCR harboring empty plasmid pJF119 as control (a). Mass ion chromatogram of (d, [1]) *para*-amino-phenylethanol (m/z 176.1 [M+K]^+^) and (e, [2]) 4- Amino phenylacetic acid (m/z 153.1 [M + +H]^+^). Chemical structure of PAPE and 4-APA present on ESI-mass spectra.


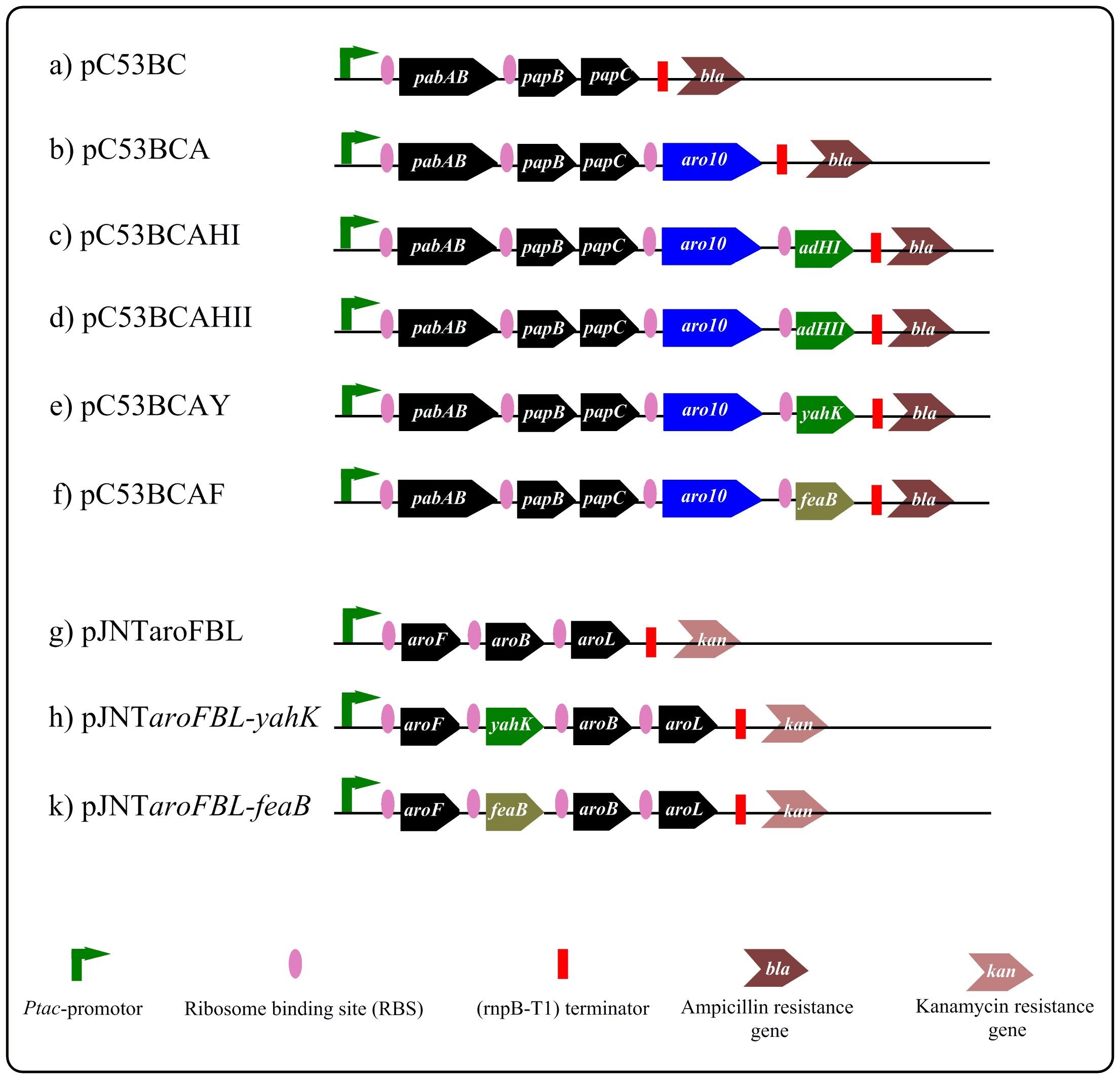


**Figure A 3.**  **Organization of the recombinant gene clusters used for production of PAPE or 4-APA in *E. coli*.** Four heterologous enzymes must be overexpressed on plasmid to mediate the synthesis of PAPE or 4-APA from chorismate. All constructs contained the *tac-*promotor and the Ribosome binding site (RBS) in front of each gene and (rnpB-T1) terminator located in the end of gene cluster.  **(a)** **pC53BC**, pJF119EH plasmid harboring PAPA genes including ***pabAB***, *p*-Amino-deoxy-chorismate synthase (ADC synthase), ***papB*** is 4-Amino-4-deoxychorismate mutase (ADC mutase) and ***papC*** is 4-amino, 4-deoxyprephenate dehydrogenase (ADC dehydrogenase) (pC53BC plasmid); **(b)** **pC53BCA**, pC53BC plasmid harboring ***aro10***, ThDP-Ketoacid decarboxylase; **(c)** **pC53BCAHI** and **(d)** **pC53BCAHII** , pC53BCA plasmid harboring **adHI (c)** and **adHII (d)**, alcohol dehydrogenase I and II; **(e)** **pC53BCAY**, pC53BCA plasmid harboring ***yahK,*** aldehyde reductase; (**f)** **pC53BCAF**, pC53BCA plasmid harboring ***feaB,*** phenylacetaldehyde dehydrogenase; **(g)** **pJNTaroFBL**, pJNT522 plasmid harboring ***aroF***- 3-deoxy-D-arabinoheptulosonate-7-phosphate-synthase (DHAP synthase), ***aroB***- 3-dehydroquinate-synthase (DHQ synthase) and ***aroL***- shikimate kinase II; (**h) pJNTaroFBL**-***yahK***, pJNTaroFBL plasmid harboring ***yahK,*** aldehyde reductase; (**k)** **pJNTaroFBL**-***feaB***, pJNTaroFBL plasmid harboring ***feaB,*** phenylacetaldehyde dehydrogenase.

**Table A1. Plasmids and strains used in this study**

| **Strains and plasmids** | **Relevant properties and genotypes** | **Source or reference** |
| --- | --- | --- |
| **Strains** |  |  |
| *E. coli* BW25113 | *lacI*^q^ *rrnB*_T14_ Δ*lacZ*_WJ16_ *hsdR*514 Δ*araBAD*_AH33_ Δ*rhaBAD*_LD78_ | (Datsenko and Wanner, 2000) |
| *E. coli* DH5α | *supE*44 Δ*lac*U169 (Ф80*lacZ*_M15), *hsdR*17 *recA*1 endA1gyrA96 thi-1 relA1 | Invitrogen |
| *E. coli* LJ110 | Wildtype W3110 (F−, λ−, IN (*rrnD-rrnE*) 1, *rph*-1) | (Zeppenfeld et al., 2000) |
| *E. coli* FUS4 | LJ110 Δ(*pheA tyrA aroF*) Δ*lac*::*Ptac*::*aroFBL* | (Gottlieb et al., 2014) |
| *E. coli* FUSBC | FUS4 Δ*tyrB::FRT,* Δ*aspC::FRT* | This work |
| *E. coli* FUSBCR | FUS4BC Δ*tyrR::FRT* | This work |
| *S.cerevisiae* W3118 | *MATa* *ura*3-52 *leu*2-3*,*112  *his*3- Δ200 *prpI::LEU*2 | (Vandenhazel et al., 1992) |
| **Plasmids** |  |  |
| pJNT-*aroFBL* | pJNT *aroF-aroB-aroL* from *E.coli* | (Mohammadi Nargesi et al., 2018) |
| pJF119EH | *P_tac_*, RBS, Amp^R^, *lacI* | (Fuerste et al., 1986) |
| pBluescript SK | Amp^R^, cloning vector | Lab stock |
| pBSK-*aro10* | pBluescript SK, *aro10* from *S. cerevisiae* W3118 | This work |
| pBSK-*ADHI* | pBluescript SK, *ADHI* from *S. cerevisiae* W3118 | This work |
| pBSK- *ADHII* | pBluescript SK, *ADHII* from *S. cerevisiae* W3118 | This work |
| pBSK-*yahK* | pBluescript SK, *yahK* from *E.coli BW25113* | This work |
| pBSK-*feaB* | pBluescript SK, *feaB* from *E.coli BW25113* | This work |
| pJFA10 | pJF119EH, *aro10* from *S. cerevisiae W3118* | This work |
| pJFA10F | pJFA10, *feaB* from *E.coli* BW25113 | This work |
| pC53BC | pJF119EH *pabAB* C.glu–*papB* and *papC* from *Streptomyces venezuelae* | (Mohammadi Nargesi et al., 2018) |
| pC53BCA | pC53BC, *aro10* from *S. cerevisiae* W3118 | This work |
| pC53BCAHI | pC53BCA, *ADHI* from *S. cerevisiae* W3118 | This work |
| pC53BCAHII | pC53BCA, *ADHII* from *S. cerevisiae* W3118 | This work |
| pC53BCAY | pC53BCA, *yahK* from *E. coli B*W25113 | This work |
| pC53BCAF | pC53BCA, *feaB* from *E. coli* BW25113 | This work |
| pJNT-*aroFBL-yahK* | pJNT*aroFBL*, *yahK* from *E. coli* BW25113 | This work |
| pJNT-*aroFBL-feaB* | pJNT*aroFBL*, *feaB* from *E.coli* BW25113 | This work |
| pKD46 | λ Red disruption system (γ, β, *exo* under control of P*araBAD*), Amp^R^ | (Datsenko and Wanner, 2000) |
| pCP20 | FLP+, λ cI857+, λ ρR Rep^ts^, Amp^R^, Cm^R^ | (Cherepanov and Wackernagel, 1995) |
| pCAS30-FRT-*cat*-FRT | Amp^R^ FRT-*cat^R^*-FRT | (Vallon et al., 2008) |

# Table A2. Primers used in this study

| **Primer Name** | **Sequences (5´-3´)** | **Restriction site (underlined)** |
| --- | --- | --- |
| *aro10*-Fw | TAAGCGGATCCTAAGGAGGAACAATATGGCACCTGTTACAATTGA | *Bam*HI |
| *aro10*-Rw | TCCGCTCTAGACTATTTTTTATTTCTTTTAAGTGCCGCTGC | *Xba*I |
| *ADHI*-Fw | CTCACCTGCAGGAAGGAGGATATACAT ATGTCTATCCCAGAAACTCAAAAAGGTGT | *Sbf*I |
| *ADHI*-Rw | TCGC GCATGCTTATTTAGAAGTGTCAACAACGTATCTACCA | *Sph*I |
| *ADHII*-Fw | ACACACCTGCAGGTAAGGAGGAACAATATGTCTATTCCAGAAACTCA | *Sbf*I |
| *ADHII*-Rw | TCCGCGCATGCTTA TTTAGAAGTGTCAACAACG | *Sph*I |
| *yahK*-Fw | CACACCTGCAGG AAGGAGGATATACAT ATGAAGATCAAAGCTGTTGGTG | *Sbf*I |
| *yahK*-Rw | TTTTGCATGC TCAGTCTGTTAGTGTGCG | *Sph*I |
| *feaB*-Fw | AGTGCCTGCAGGAAGGAGGATATACATATGACAGAGCCGCATGT AGCAG | *Sbf*I |
| *feaB*-Rw | AGAGCATGCTTAATACCGTACACACACCGA | *SphI* |
| Del-*aspC*-fw | GTGTTTCAAAAAGTTGACGCCTACGCTGGCGACCCG AGCTTGCATGCAGATTGCAGC | - |
| Del-*aspC*-rev | TTACATCACCGCAGCAAACGCCTTTGCCACACGTTG AGCTTAACGGCTGACATGGGA | - |
| Del-*tyrB-*fw | ATGTTTGAGAACATTACCGCCGCTCCTGCCGACCCG AGCTTGCATGCAGATTGCAGC | - |
| Del-*tyrB*-rev | TTACAGCACTGCCACAATCGCTTCGCACAGCGGAGC AGCTTAACGGCTGACATGGGA | - |
| Ko-*aspC*-fw | TCCAGAGCAATCTCACGTCTT | - |
| Ko-*aspC*-rev | C ACGAGCGCCT TATCCGGCCT | - |
| Ko-*tyrB*-fw | GGATGTACGT TTGTCATGAG T | - |
| Ko-*tyrB*-rev | CTCTCACGTAGAACGATGGCA | - |

**Reference**

Cherepanov, P.P., and Wackernagel, W. (1995). Gene disruption in *Escherichia coli*: TcR and KmR cassettes with the option of Flp-catalyzed excision of the antibiotic-resistance determinant. *Gene* 158**,** 9-14.

Datsenko, K.A., and Wanner, B.L. (2000). One-step inactivation of chromosomal genes in *Escherichia coli* K-12 using PCR products. *Proc Natl Acad Sci U S A* 97**,** 6640-6645.

Fuerste, J.P., Pansegrau, W., Frank, R., Blocker, H., Scholz, P., Bagdasarian, M., and Lanka, E. (1986). Molecular cloning of the plasmid RP4 primase region in a multi-host-range tacP expression vector. *Gene* 48**,** 119-131.

Gottlieb, K., Albermann, C., and Sprenger, G.A. (2014). Improvement of L-phenylalanine production from glycerol by recombinant *Escherichia coli* strains: the role of extra copies of *glpK*, *glpX*, and *tktA* genes. *Microb Cell Fact* 13**,** 96.

Mohammadi Nargesi, B., Trachtmann, N., Sprenger, G.A., and Youn, J.W. (2018). Production of *p*-amino-L-phenylalanine (L-PAPA) from glycerol by metabolic grafting of *Escherichia coli*. *Microb Cell Fact* 17:149.

Vallon, T., Ghanegaonkar, S., Vielhauer, O., Muller, A., Albermann, C., Sprenger, G., Reuss, M., and Lemuth, K. (2008). Quantitative analysis of isoprenoid diphosphate intermediates in recombinant and wild-type *Escherichia coli* strains. *Appl Microbiol Biotechnol* 81**,** 175-182.

Vandenhazel, H.B., Kiellandbrandt, M.C., and Winther, J.R. (1992). Autoactivation of Proteinase-a Initiates Activation of Yeast Vacuolar Zymogens. *Eur J Biochem* 207**,** 277-283.

Zeppenfeld, T., Larisch, C., Lengeler, J.W., and Jahreis, K. (2000). Glucose transporter mutants of *Escherichia coli* K-12 with changes in substrate recognition of IICB(Glc) and induction behavior of the *ptsG* gene. *Journal of bacteriology* 182**,** 4443-4452.
